# Organ Segmentation in Poultry Viscera Using RGB-D

**DOI:** 10.3390/s18010117

**Published:** 2018-01-03

**Authors:** Mark Philip Philipsen, Jacob Velling Dueholm, Anders Jørgensen, Sergio Escalera, Thomas Baltzer Moeslund

**Affiliations:** 1 Media Technology, Aalborg University, 9000 Aalborg, Denmark; jvdu@create.aau.dk (J.V.D.); andjor@create.aau.dk (A.J.); sergio.escalera.guerrero@gmail.com (S.E.); tbm@create.aau.dk (T.B.M.); 2 IHFood, Carsten Niebuhrs Gade 10, 2. tv., 1577 Copenhagen, Denmark; 3 Mathematics and Informatics, University of Barcelona, 08007 Barcelona, Spain; 4 Computer Vision Center, Bellaterra, 08193 Barcelona, Spain

**Keywords:** semantic segmentation, RGB-D, random forest, conditional random field, 2D, 3D, CNN

## Abstract

We present a pattern recognition framework for semantic segmentation of visual structures, that is, multi-class labelling at pixel level, and apply it to the task of segmenting organs in the eviscerated viscera from slaughtered poultry in RGB-D images. This is a step towards replacing the current strenuous manual inspection at poultry processing plants. Features are extracted from feature maps such as activation maps from a convolutional neural network (CNN). A random forest classifier assigns class probabilities, which are further refined by utilizing context in a conditional random field. The presented method is compatible with both 2D and 3D features, which allows us to explore the value of adding 3D and CNN-derived features. The dataset consists of 604 RGB-D images showing 151 unique sets of eviscerated viscera from four different perspectives. A mean Jaccard index of 78.11% is achieved across the four classes of organs by using features derived from 2D, 3D and a CNN, compared to 74.28% using only basic 2D image features.

## 1. Introduction

Poultry is both the most popular and the fastest rising source of meat in the USA [1]. The number of slaughtered chickens sold to US consumers was 8.6 billion in 2010, with the US broiler industry valued at $45 billion [2]. The health inspection at poultry processing plants involves visual inspection of the viscera, that is, the internal organs of the chicken, after it has been extracted from the carcass. The inspection is necessary in order to ensure that the evisceration has been successfully performed and to examine whether the chicken shows any signs of disease. Hearts and livers are sold for human consumption. Therefore, it is important that these organs are extracted undamaged and show no signs of disease. Visual abnormalities such as inflammation makes organs such as liver and heart, if not a health risk, then unappetizing to look at. Incomplete removal of viscera is a quality issue in chickens that are sold whole.

The current process for inspecting the viscera involves strenuous manual labour, which limits the throughput of the processing plant. The operators must inspect approximately three chickens per second. In addition to inspecting the viscera and corresponding carcasses, the birds that fail the inspection must be manually removed from the production line. Manual inspection is inherently slow, expensive and inconsistent; all of which represent a challenge when attempting to comply with the increasing demands from consumers.

Machine vision has previously been explored for inspection and grading of poultry. A great introduction to the problem and existing work on vision-based poultry inspection is given in [3], where a general-purpose multispectral classification system for high-speed online inspection of chicken carcasses is presented. In [4], a hyper-spectral imaging system for detection of external faeces and ingesta is proposed. External inspection has been commercially available for some time, for example, with the ClassifEYE [5] grading system for detecting damaged and impure carcasses. Inspection of the viscera, on the other hand, has received less attention. The most recent example of work in this area is [6], where entire poultry viscera are classified into four categories, that is, normal, airsacculitis, cadaver and septicemia, using a neuro-fussy model of RGB and spatial features. This model is able to classify the viscera correctly 82.5% of the time. In this paper, we take a different approach as the viscera is first segmented into the organs of interest. This segmentation will then serve as the basis for the subsequent work that deals with detection of disease and quality control. As an example, in [7], the segmentation approach described in our previous work [8] is used as the basis for classifying patches of liver as healthy or according to different diseases. The organs that are of interest during inspection are the heart, liver and lungs. The rest of the viscera consists of the intestines, minor organs and connective tissue; all of which are to be classified together. Viscera are non-rigid bodies without straight lines or sharp edges and obey only a weak spatial arrangement. The nature of these objects are thus very different from the data that most object segmentation research deals with.

With this work, we exploit the recent advances in computer vision and pattern recognition by applying a modern visual perception framework to an unusual dataset in a problem domain that has seen limited progress in the last decade. We make use of a convolutional neural network (CNN) and 3D-derived features and demonstrate their contribution to the overall performance of the semantic segmentation of viscera. This paper is an extension of the work in our original conference paper [8]. The extended work includes four times the amount of data, CNN features and the addition of a conditional random field (CRF) on top of the random forest (RF) classifier. These improvements, in combination, lead to an increase in the mean pixel-wise Jaccard index from the 61.5% achieved in [8] to the 78.11% achieved here.

### Contributions

This work can be summarized as: (1) using CNN activation maps as superpixel features; (2) quantifying the value of CNN- and 3D-derived features for semantic segmentation; (3) proposing a method for visual localization of organs enabling automated quality control.

## 2. Related Work

Quality control of organic material is based largely on hyper-spectral imaging (HSI). HSI makes it possible to capture nuances in colors that are normally not visible with RGB cameras [9,10,11,12,13]. In [14], splenomegaly is detected on poultry carcasses using ultraviolet (UV) and color imaging. The use of UV aids in separating the spleen from the liver; something that proves difficult in RGB images. In [15], it is concluded that near-infrared imaging can be used to access quality measures like tenderness and color of fresh beef. Reference [16] investigated 33 wavelengths in the visible spectrum and discovered two wavelengths, namely 600 nm and 720 nm, that are optimal for detecting the gallbladder attached to the chicken liver.

More complex approaches with segmentation of multiple organs are less explored. An example is found in [17], where RGB images of pig offal are segmented into five classes (heart, liver, lungs, diaphragm and an upper portion). This is achieved with a modified auto-context algorithm with an updating atlas, showing a small yet consistent improvement over the regular auto-context algorithm. A comparable problem domain is medical machine vision, for example, the analysis of magnetic resonance brain scans. Given the relatively rigid structure of the brain, atlas-based approaches have proven successful in deforming expert-annotated samples to the target image. Multiple atlases can be used to increase the accuracy at the expense of higher computational cost [18,19]. Reference [20] compares an atlas-based approach to a tree-based approach using both computed tomography and magnetic resonance scans. A multivariate regression forest is used to predict the 3D bounding volumes of multiple organs based on appearance, spatial context and confidence prediction. The tree-based model was found to be more efficient than the atlas-based approaches, noting that tree-based methods implicitly contain a prior assumption of spatial information. The tree-based models are preferred in this application due to the weak spatial structure. Generalized models with little to no prior assumptions have also recently been proposed for various medical segmentation tasks suitable for a wide range of modalities [21]. Based on standard 3D features, they show improvements on three medical volume data sets using an iterative learning scheme of stacked contextual classifiers, where each stacked classifier adds complexity.

Most existing research in semantic scene segmentation is focused on scenes with man-made objects. Contrary to the organs in our viscera dataset, man-made objects comprise, to a large extent, straight lines and clean edges. Two widely used datasets with these types of objects are NYU-D 1 [22] and NYU-D 2 [23]. They both contain RGB and depth (RGB-D) images of indoor scenes. Reference [24] is one example of recent work that addresses the NYU-D V2. They apply a framework, where features are extracted from superpixels and classified using an RF. The label predictions are then refined using a CRF, which applies a pairwise smoothness term and learns contextual relationships between the different classes. A similar approach is found in [25]; instead of operating directly on the image channels of the RGB-D image, a point cloud is the basis for the oversegmentation and feature extraction. A similar approach is seen in [26] where a pixel-wise RF with random offset features and a CRF are expanded by using a stacked random forest. The stacked classifier learns when the previous classifier is mistaken and attempts to correct the errors.

Deep-learning architectures for semantic pixel-wise segmentation have, as in many other areas, raised the bar significantly from the former approaches that relied on handcrafted features and classic classifiers. The VGG CNN architecture [27] has proven very versatile. Even though it was originally intended to address the image classification problem, it now forms the basis of many of the deep-learning approaches that attempt to solve dense semantic segmentation datasets. Reference [28] shows that the VGG architecture outperforms AlexNet [29] and GoogLeNet [30] when using its weights in their fully convolutional network (FCN). This is the first FCN to be rearchitected from pre-trained classification networks, such as AlexNet [29], VGG [27] and GoogLeNet [30]. The rearchitecting process is primarily done by discarding the final classification layer, converting the fully connected layers to convolutional layers and appending 1 × 1 convolutions in order to predict class scores at each coarse output location. By fine-tuning the entire pre-trained set of weights they gain a 30% better performance compared to only fine-tuning the output classifier. Reference [31] proposes a new FCN architecture for semantic pixel-wise segmentation, where the low-resolution feature maps produced by the encoding convolutional layers of the VGG model are upsampled to input image resolution by passing max-pooling indices from the encoding part of the network to the corresponding decoding layers. Finally, their architecture produces probability maps for each of the classes. The performance of their method is evaluated on the CamVid road scene segmentation dataset, the PASCAL VOC 2012 dataset and the SUN RGB-D indoor scene understanding dataset [32]. They compare their approach to several other deep-learning and non-deep-learning approaches, and they mostly achieve state-of-the-art performance along with the lowest computational cost. Again, in [33], the pre-trained weights of the VGG network are used in another adaption of a CNN to semantic image segmentation. They overcome the problems with the loss of spatial information associated with the use of CNNs by replacing downsampling operators in the final max-pooling layers and upsampling the filters in subsequent convolutional layers. The score maps are then upsampled to input resolution using bilinear interpolation and passed to a fully connected CRF, which captures fine-edge details and long-range dependencies. In [34], the authors propose to use the activations from across multiple layers of a CNN to describe pixels and perform fine-grained semantic segmentation. The early layers contain low-level information with a high localization precision, while the later layers capture high-level semantic information that is much less sensitive to pose and placement. The combination of both types of features results in pixel-wise feature vectors that allow for high localization precision and great semantic classification.

## 3. Chicken Viscera Dataset

**Figure 1:**
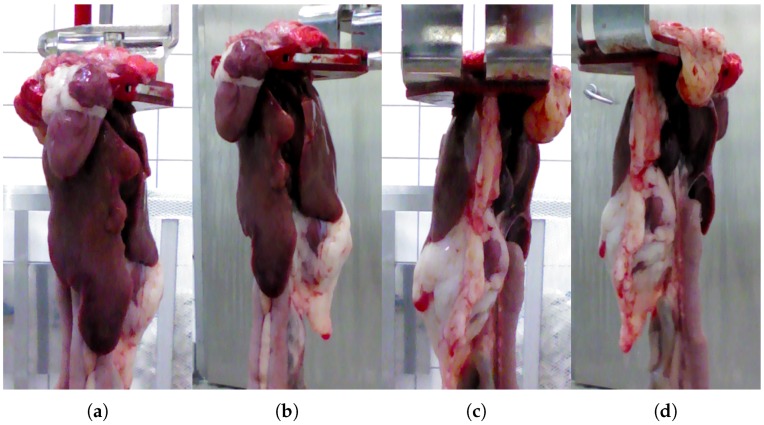
A single set of viscera as presented on the production line. Each set is captured from four perspectives. (**a**) Center front; (**b**) right front; (**c**) center back; (**d**) right back.

A dataset consisting of 151 sets of chicken viscera was collected for our previous work [8]. Each set is captured from four different perspectives as shown in Figure 1, resulting in a total of 604 RGB-D images. The viscera were captured from a distance of 35 cm and placed in a hanger similar to the ones used on the production line. The viscera were taken directly from the production line and placed in the hanger, while retaining the same orientation as on the line.

**Figure 2:**
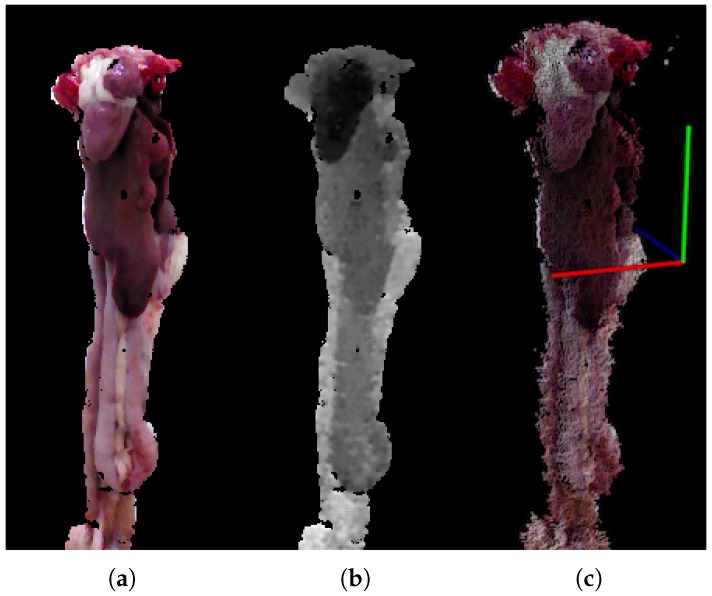
(**a**) RGB; (**b**) depth; (**c**) point cloud.

Data is captured using the RealSense F200 3D camera and the RealSense SDK [35] from Intel, USA. The RGB images are registered to the depth maps, using the calibration provided by the Intel RealSense SDK, resulting in RGB-D images with a resolution of 480×640 pixels with an approximate region of interest of 150×350 pixels. Figure 2 shows the different representations of the RGB-D input data, specifically the RGB image Figure 2a, the depth image Figure 2b and a point cloud Figure 2c.

### Ground-Truth Annotations

**Figure 3:**
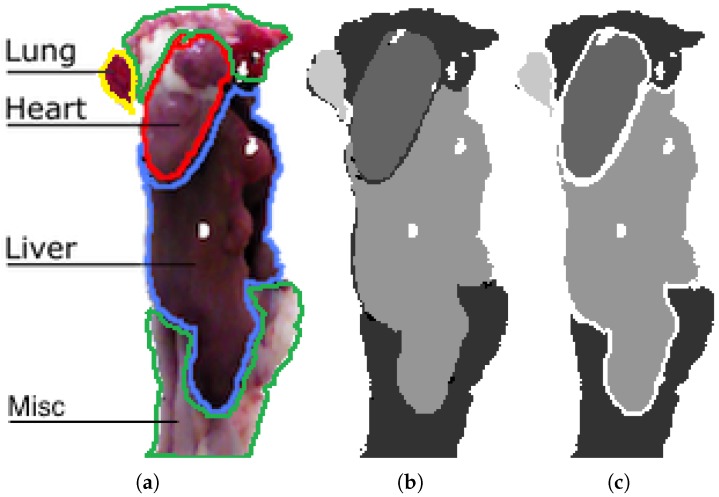
(**a**) RGB image of entrails with labeled organs; (**b**) manual annotation in 2D; (**c**) manual annotation with ignore region used onwards in this work. The labels indicate with increasing intensity: miscellaneous, heart, liver and lung.

A ground-truth (GT) is needed to train and evaluate supervised learning algorithms. For this dataset, the GT is obtained by manually annotating pixels belonging to the three classes of interest, namely heart, liver and lungs, and an additional fourth category encompassing miscellaneous organs and tissue. An ignore region is established around each organ, as labelling data is an ill-posed problem where the assignment of labels near borders is ambiguous. Figure 3a shows an example of a viscera in RGB with the four classes lined out. Figure 3b shows the corresponding grayscale pixel-level annotation and Figure 3c shows the same annotation including a 2-pixel ignore boundary around each class. The ignore region furthermore contains pixels with no depth measurement, which can be seen as holes in the image. Pixels in the ignore region are excluded from both training and evaluation. The use of ignore regions is also seen in the popular pascal segmentation competition [36]. Ignore regions are introduced to avoid penalizing systems because of ambiguities in the labelling of pixels. At the same time, these are the areas that are the most challenging for semantic segmentation systems, thus ignoring them will unavoidably lead to improvements in the performance metrics. The specific width of the ignore regions used on this dataset was the result of discussions with end-users. Because of the significant size difference for various organs and occlusion, the number of pixels for each class is skewed with 5% heart, 20% liver, 5% lung and 70% miscellaneous.

Unlike in our preliminary work [8], where only 151 images from a single perspective were used, this work makes use of all 604 images. Since the same viscera recur multiple times in the data set, although from different viewpoints, it is emphasized that images of the same viscera do not appear across the training and test sets.

## 4. Segmentation Approach

**Figure 4:**
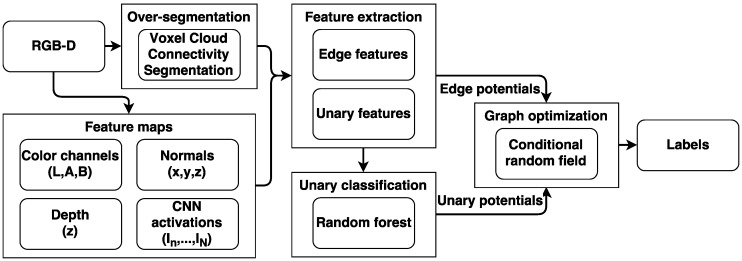
Overview of the segmentation framework.

In this paper we apply a framework for semantic segmentation inspired by the work of Müller [24] and Wolf [25]. The framework, as shown in Figure 4, consists of oversegmenting the point cloud into supervoxels, from where features are extracted from feature maps, which are used to assign class probabilities in the RF classifier. A CRF optimizes the label assignments by taking the similarities between neighbors into account. The funding program supporting this work aims at demonstrating concepts in a collaboration between end-users, companies and one or more universities. Issues such as computational, memory and speed requirements are normally not included in such a project. Instead, a proof of concept is the target. In case of a successful project, the idea is that the company continues to mature the technology. We have therefore not been focusing on implementation issues and used standard C++ and Python libraries whenever possible. Our current implementation is therefore not real-time. The used methodology is tailored to the specific type of dataset, in particular the very small dataset size and the organic objects. The presented method will not perform favorably when compared to the end-to-end deep-learning-based techniques that dominate large public RGB-D semantic segmentation benchmarks. Likewise, these state-of-the-art methods are difficult to get to perform well on such a specialized and limited dataset. The novelty of our framework lies in the use of CNN features and their application on an unusual dataset, which leads to the omission of features commonly used addressing man-made objects. 

Both the RF and the CRF are supervised classification methods that require training on labelled data. The dataset of 151 viscera sets are split into an RF training set of 91 sets, a CRF training set of 30 sets, while the remaining 30 sets are reserved for testing.

### 4.1. Oversegmentation

Oversegmentation is often used as a pre-processing step, where similar pixels are grouped into superpixels in the 2D case [37], or supervoxels in 3D [38]. Oversegmentation reduces the computational complexity at the later stages and can help reduce noise due to small variations, at the expense of a loss in precision depending on the oversegmentation’s boundary adherence.

In this framework the voxel cloud connectivity segmentation (VCCS) [38] supervoxel segmentation algorithm is used, as the importance of each feature type that is used for oversegmentation, namely color, spatial and geometric, can be adjusted using weights. This is convenient for exploring the value of features derived from 3D. VCCS produces the supervoxels by seeding the point cloud spatially evenly and an iterative clustering algorithm groups voxels within $\sqrt{3}R_{seed}$ of each seed in a 39-dimensional space based on spatial, color and geometric similarity.

### 4.2. Feature Maps

**Figure 5:**
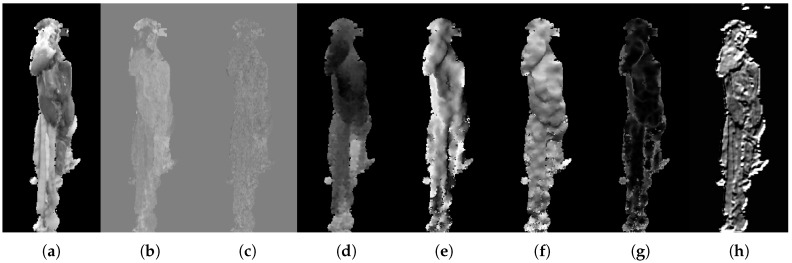
Feature map examples. (**a**) Channel L from LAB; (**b**) channel A from LAB; (**c**) channel B from LAB; (**d**) depth map; (**e**) normal magnitude in *x* direction; (**f**) normal magnitude in *y* direction; (**g**) normal magnitude in *z* direction; (**h**) CNN activation map.

Feature maps are obtained in preparation for the feature extraction and include the color channels from the LAB space, depth, normal magnitude for each of the three dimensions, and activation maps from across different layers of a CNN. Examples of each type of feature map are shown in Figure 5. The viscera is subject to a loose composition, where heart and lungs are generally found in the top while the liver usually configures in the central or bottom part of the image. Therefore, the position of a superpixel is a valuable feature. The CNN activation maps are extracted from each filter of each convolutional layer in the VGG-16 architecture [27]. From all of the convolutions layers, a total of 4224 CNN feature maps are produced. The used network weights originate from the network that was trained as part of the VGG team’s entry to the ILSVRC-2014 competition and are used directly without fine-tuning. The specifics of the network are described in [27]. The activation maps that are lower resolution than the input are simply resized using linear interpolation. This use of CNN feature maps is inspired by the work of [34], where it is shown that extracting information at various layers across the network helps preserving the localization information present in the lower layers. This is done by creating pixel-wise feature vectors across the layers.

### 4.3. Feature Extraction

The features used for classification are extracted for each superpixel. The feature responses for each feature map are averaged for all pixels in a superpixel. Two sets of features are extracted. Unary features are extracted for the RF, and secondly, pairwise features are found for the CRF.

Unary features are used in the RF to assign probabilistic labels to each superpixel. These probabilities serve as the unary component of the optimization performed later by the CRF. The features that are useful here are good at discriminating between the classes. The unary features used in the RF are listed in Table 1.

Edge features are used to measure the similarity of neighboring superpixels and serve as the pairwise component of the CRF. The features that are used here must be good at describing the similarity of two superpixels. The edge features for the CRF are listed in Table 1.

### 4.4. Unary Classification

The unary potentials are used as an initial label estimate for the superpixels. Because the classification of the unary features is based entirely on local features, and since the neighborhood is taken into account at a later stage, these labels are assigned with a probability instead of one-hot encoding. Thereby, the later optimization can take into account the uncertainty of the initial classification. In this work the RF classifier [39] is used for the initial classification. The RF consists of an ensemble of label distributions in the leaf node trees. The label distributions are created, during training, from labelled training features that reach a particular leaf node when traversing through the RF.

**Figure 6:**
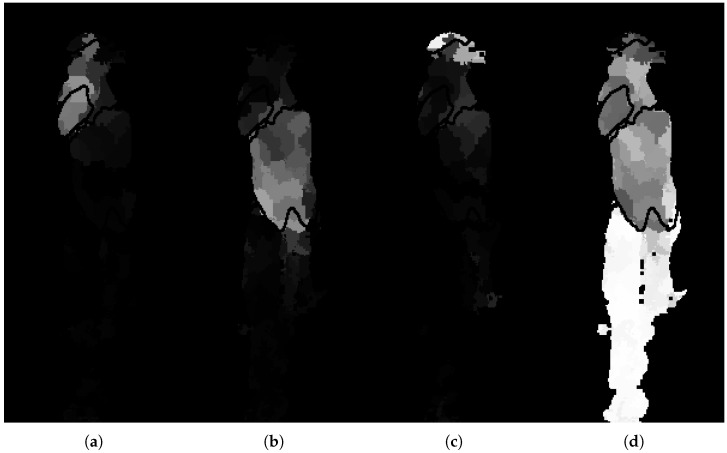
Random forest probabilities for each superpixel belonging to each of the four classes. (**a**) Heart; (**b**) liver; (**c**) lung; (**d**) miscellaneous. White values indicates higher certainty from the classifier.

The RF is implemented using the scikit-learn library [40]. The dataset is imbalanced due to the differences in organ size and occurrence. In order to compensate for this, prior probabilities that reflect the skewed distribution are assigned to each class. The number of trees in the forest is determined by examining the convergence of the out-of-bag error. The remaining parameters of the RF are optimized through a cross-validated grid search on 91 sets of viscera, reserved for training the RF. The class probabilities produced by the trained RF model are visualized with the example in Figure 6. It is clear that the unary potentials give a rough location and segmentation for each organ. The model is certain for a few superpixels, especially for the miscellaneous part found in the bottom half of the image, as seen in Figure 6d. The heart is less obvious with several faulty responses found on the liver. Using these local unary potentials alone and based on a hard-decision scheme where the most likely outcome is chosen across all classes, as seen in Figure 6c, results in large parts of the liver being misclassified as miscellaneous.

### 4.5. Graph Optimization

The random forest prediction based on local features can be further improved by taking neighborhoods into account. This is done using a CRF [41], where the learned edge potentials describe the likelihood of relationships between the different classes, according to the composition of the edge features between them.

**Figure 7:**
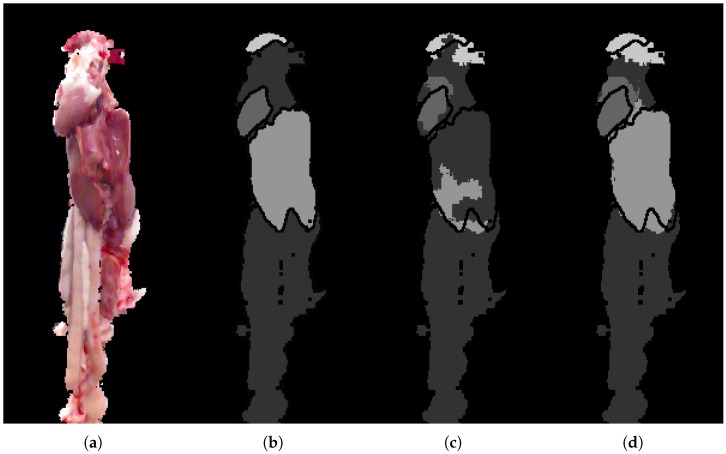
Final prediction where labels are, with increasing intensity: miscellaneous, heart, liver and lung. (**a**) input image; (**b**) GT; (**c**) random forest prediction using 3D and CNN features; and (**d**) RF + CRF using 3D and CNN features.

The optimization that the CRF performs is based on the minimization of the energy function consisting of unary potentials and pairwise potentials. The unary potentials originate from the class label probabilities of the RF classifier. In this work the pairwise features consist of difference in position and color information, and normals in the 3D case, all learned from the separate training set. Note that each class is weighted according to the inverse class frequency. The CRF is implemented using the freely available PyStruct [42]. It is shown to have a smoothing effect as seen in Figure 7, refining the RF classification by a large portion of especially the liver in this case. The CRF is able to correctly infer superpixels even over longer distances given certain unary potentials and discriminative pairwise potentials.

## 5. Evaluation

The value of adding 3D and CNN features is documented by progressively adding the different feature types to the semantic segmentation pipeline and evaluating the performance. The evaluation is based on 120 images displaying 30 unique viscera from different angles. Evaluation is performed at the pixel level. The regions with background and the 2-pixel-wide boundary area around classes are excluded.

### 5.1. Quantitative Analysis

The segmentation is scored by the Jaccard index shown in (1), measuring the similarity between the prediction and the manually labeled GT by counting true positives (tp), false positives (fp) and false negatives (fn). The evaluation is performed on the pixel level, where every pixel belonging to a given superpixel inherits the superpixel’s label.

(1) $$Jaccard\;index=\frac{tp}{tp+fp+fn}$$

The Jaccard index is found for each class and averaged, without weighting according to class occurrence, for a combined score. For the results based exclusively on 2D features, the features that are related to 3D are disabled. This is impacting the clustering into superpixels, as the geometric similarity is an important feature, as well as the RF classification from the lack of depth and the CRF from the lack of normal features. The additional information of both 3D and CNN is shown to improve the segmentation as seen in Table 2, especially on the more challenging heart class. The heart is small and much of it is covered by fat, which makes the color features much less effective. The lungs are also small and often occluded, therefore there are fewer pixels available for training and testing. Additionally, the lungs exhibit large variance in color, based on the amount of blood left in them.

The use of superpixels results in an upper limit on overall accuracy, which depends on the object boundary adherence of the oversegmentation. The impact of the oversegmentation is measured using an upper bound, namely the achievable segmentation accuracy (ASA) [43] defined in (2). Each superpixel/supervoxel $S_{k}$ in S is labeled with the ground-truth label $G_{i}$ with the largest intersection, and finally normalized where a score of 1 is a perfect fit. The maximal performance with the employed oversegmentation is show under ASA in Table 2.

(2) $$ASA_{G}\left(S\right)=\frac{\sum_{k}\max_{i}\left|S_{k}\cap G_{i}\right|}{\sum_{i}\left|G_{i}\right|}$$

**Figure 8:**
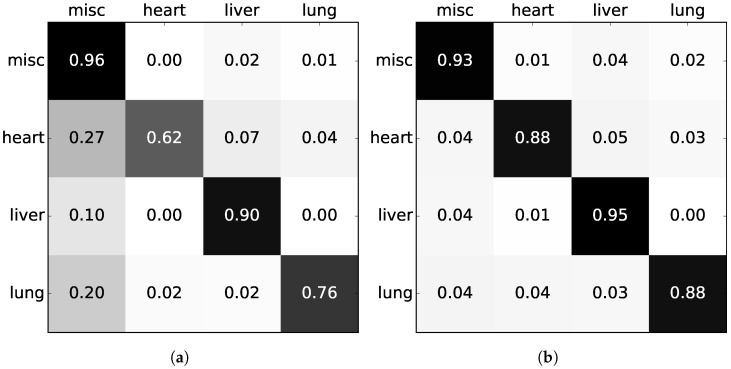
Normalized confusion matrices with the true label on the left and prediction on top. (**a**) RF with 3D + CNN features; (**b**) RF + CRF with 3D + CNN features.

The confusion matrices of Figure 8 have been inspected to gain insight into the errors being made. The confusion matrices are normalized to account for the imbalanced dataset. The RF has a tendency to favor the miscellaneous class, with several misclassifications among all the other three classes. The CRF, on the other hand, is a more balanced system with evenly distributed errors.

### 5.2. Analytic Results

**Figure 9:**
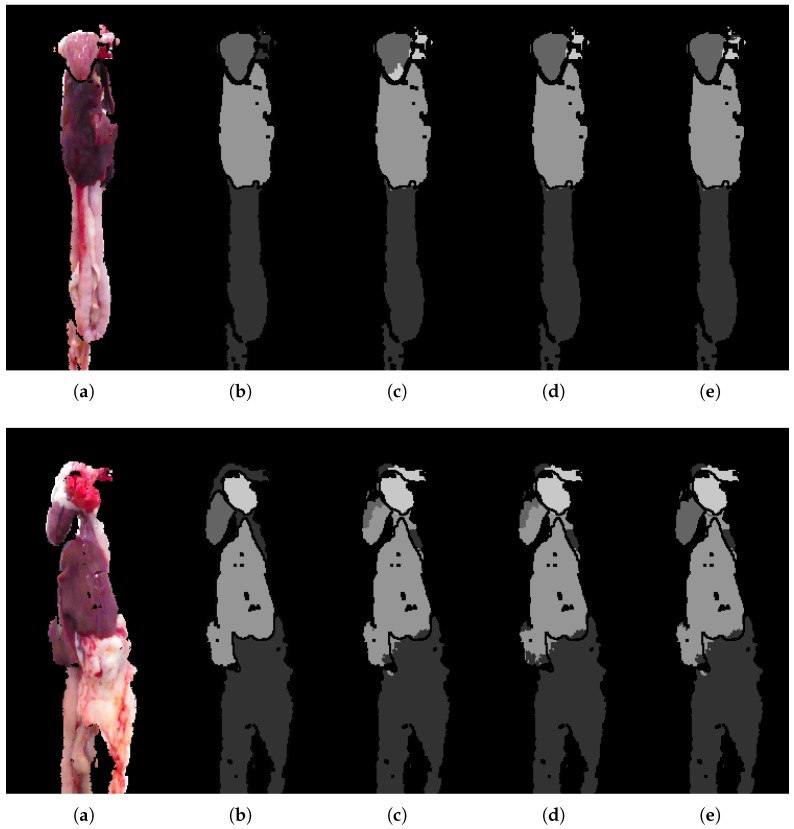

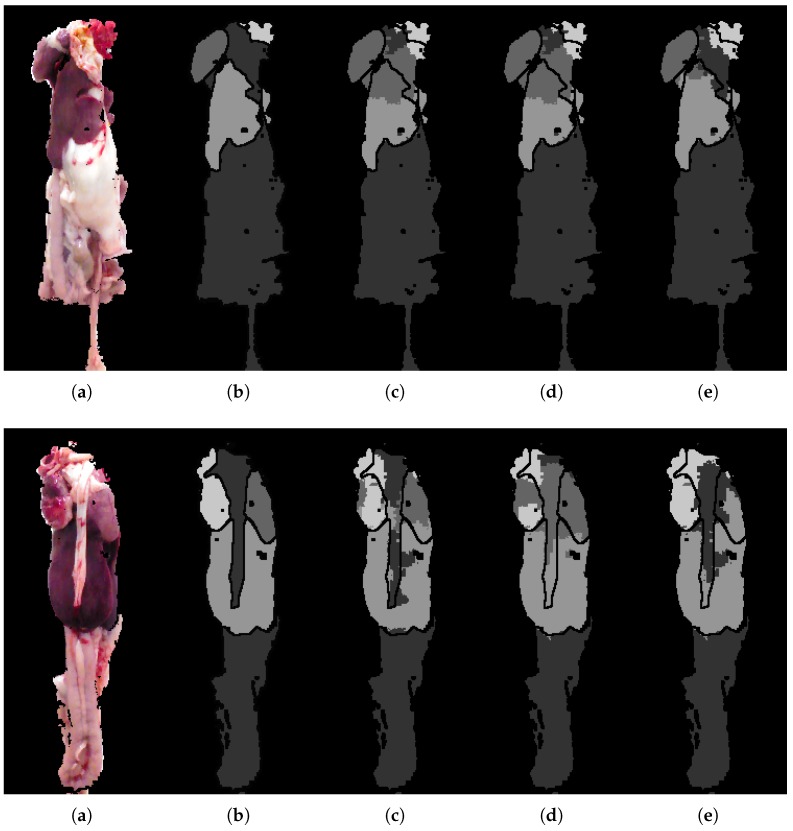
Examples for qualitative analysis. (**a**) RGB image; (**b**) ground-truth; (**c**) RF + CRF + 2D; (**d**) RF + CRF + 3D; (**e**) RF + CRF + 3D + CNN.

The examples shown in Figure 9 are included to visualize the improvements shown in the quantitative analysis. Even though it is limited to a few percentage points, the improvement is visually evident, and can ultimately mean the difference between correctly segmenting, for example, a heart or not, as seen in the second row. The heart and lung classes are very small in terms of pixels. For this reason a small improvement in the precision of the unary classification may lead to a large jump in overall performance, especially if it leads to a small region of the heart to suddenly be found, allowing the CRF to get a better starting point for optimizing and correcting neighboring regions. The segmentation is found to be most challenged in the top where lung, heart and miscellaneous are found in close proximity. On the other hand, segmentation of liver and miscellaneous in the lower regions of the images is proving relatively robust. The RF seems to struggle in areas with high variance, resulting in almost arbitrary class predictions. This uncertainty is seen to traverse through the framework, complicating the task of the CRF. The CRF is highly dependent on the input from the RF, and thereby also the features used. Depending on the application, it is desirable to get somewhat continuous regions of the same label, that is, the segmentation produces a single coherent region for the heart and another with liver, and so on. The CRF smoothing effect is found to overall improve the segmentation and produce more desirable regions.

## 6. Conclusions

We presented a framework for automatic visual inspection of poultry viscera, a domain with a weak spatial arrangement without sharp edges and with organs often occluded by fat. We show how segmentation algorithms previously used on man-made objects are able to function on these deformable objects, scoring a mean Jaccard index of 74.28% across the four classes of organs by using basic 2D features. The addition of 3D features shows a potential improvement, with a score of 76.03% despite only small depth deviations of the viscera. Finally, adding CNN features achieves a mean Jaccard index 78.11%.

## Figures and Tables

**Table 1:** Unary and edge features.

Unary Features	Type	2D	3D	3D + CNN
LAB	Color	3	3	3
Center point	Spatial	2	3	3
CNN activation	Texture etc.	0	0	4224
**Edge Features**	**Type**	**2D**	**3D**	**3D + CNN**
LAB	Color	3	3	3
Center point	Spatial	2	3	3
Normal vector	Geometric	0	3	3

**Table 2:** Pixel-wise Jaccard index for the four classes when evaluating using a 2-pixel-wide ignore region.

Method	Features	Misc.	Heart	Liver	Lung	Class Avg.
RF + CRF	2D	90.66	57.69	80.59	68.18	74.28
RF + CRF	3D	91.28	63.02	82.38	67.43	76.03
RF + CRF	3D + CNN	91.58	70.17	83.64	67.05	78.11
ASA		96.32	88.65	88.63	82.49	89.63
